# Rice Domestication Revealed by Reduced Shattering of Archaeological rice from the Lower Yangtze valley

**DOI:** 10.1038/srep28136

**Published:** 2016-06-21

**Authors:** Yunfei Zheng, Gary W. Crawford, Leping Jiang, Xugao Chen

**Affiliations:** 1 Zhejiang Provincial Institute of Cultural Relics and Archaeology, Jiashan Road, Hangzhou, 310014, China; 2 Department of Anthropology, University of Toronto Mississauga, Mississauga, Ontario, L5L 1C6, Canada

## Abstract

Plant remains dating to between 9000 and 8400 BP from a probable ditch structure at the Huxi site include the oldest rice (*Oryza sativa*) spikelet bases and associated plant remains recovered in China. The remains document an early stage of rice domestication and the ecological setting in which early cultivation was taking place. The rice spikelet bases from Huxi include wild (shattering), intermediate, and domesticated (non-shattering) forms. The relative frequency of intermediate and non-shattering spikelet bases indicates that selection for, at the very least, non-shattering rice was underway at Huxi. The rice also has characteristics of japonica rice (*Oryza sativa* subsp. *japonica*), helping to clarify the emergence of a significant lineage of the crop. Seeds, phytoliths and their context provide evidence of increasing anthropogenesis and cultivation during the occupation. Rice spikelet bases from Kuahuqiao (8000–7700 BP), Tianluoshan (7000–6500 BP), Majiabang (6300–6000 BP), and Liangzhu (5300–4300 BP) sites indicate that rice underwent continuing selection for reduced shattering and japonica rice characteristics, confirming a prolonged domestication process for rice.

Archaeological evidence for the initial steps leading to domesticated rice (*Oryza sativa*) in China is elusive despite being better informed recently by genetic, archaeological, palaeoenvironmental, and archaeobotanical data^1^.While phytoliths from the Shangshan site indicate that rice domestication was underway some time between 12,500 and 7500 BP^2^. Phytoliths are silent on two questions: 1) when did key domestication syndrome traits, particularly non-shattering spikelets start undergoing selection by people, and 2) under what conditions did the traits develop? A third issue, the circumstances and timing of japonica rice (*Oryza sativa* subsp. *japonica*) differentiation are also problematic. Complementary data sets comprised of rice spikelet bases and seeds of a range of taxa recovered by a form of wet screening, and phytoliths from a stratified structure at the Huxi site (9000–8400 BP) address these questions for the first time. Huxi belongs to the Shangshan Culture, the oldest culture so far documented in the lower Yangtze basin. Previous research indicates that rice domestication was well under way by 8000–5500 BP^3^,^4^,^5^ and that paddy field engineering was established between 7000 and 4000 BP^6^,^7^,^8^. Other domesticated organisms such as pig and peach were managed in a landscape that was undergoing anthropogenic transformation by 7600 BP^9^.

Rice husks contained in pottery matrices from the Shangshan site (ca. 10,000 BP)^10^,^11^ stimulated a heated debate on the origin of domesticated rice^12^,^13^,^14^,^15^. The debate was triggered by a preliminary observation of grain length/width ratios hinting that the rice embedded in the Shangshan site pottery was an early domesticated type. Preserved organic matter, charred or otherwise preserved, has until now been rare at Shangshan culture sites, mainly appearing as inclusions in pottery matrices with few spikelet bases being evident. As such, a more comprehensive analysis of the rice from Shangshan has been difficult to achieve, particularly since rice spikelet bases are key to understanding the nature of rice selection^16^.

The Shangshan culture Huxi site (28°52′28.27“N, 120° 1′4.58“E) (Fig. 1) is an open-air site in Yongkang County, Zhejiang Province, China, dating 9000–8400 BP (Fig. 2, Supplementary Table S1). Huxi is situated in a flat basin about 100 m above sea level, surrounded by low hills and about 2 km from the Yongkang River. A 1.5 m deep cultural deposit in what was possibly a ditch that filled with sediment and cultural debris over time is divided into six strata (Supplementary Fig. S1). Several pits extended into otherwise undisturbed sediment that forms the floor of the potential ditch. The artifact density is high and contains well-made, thin-walled pottery decorated with red slip, stone querns, and stone balls. Animal bones, charcoal, and plant seeds are also present due to relatively anaerobic conditions in the deepest deposits.

This paper reports the earliest rice remains and associated plant macro- and micro-fossils so far recovered from archaeological sediment samples in the Yangtze Valley, and assesses this assemblage in order to understand both the status of rice domestication and its human ecological context at the time. In particular, we address the development of non-shattering spikelets by examining the variation of abscission layer development at the cellular level in archaeological rice spikelet bases for the first time. The rice remains are assessed in the context of the divergence of japonica (*O. sativa* subsp. *japonica*) and indica (*O. sativa* subsp. *indica*) rice. Current genetic research points to japonica arising first from a progenitor population that was already diverging in the japonica direction and that indica evolved as a result of hybridization of domesticated japonica with local rice in South Asia^17^,^18^,^19^.

## Results

### Phytoliths

All samples contain high densities of Poaceae bulliform phytoliths (from leaf epidermis) and bi-peaked tubercles from rice husks (palea and lemma).

**Figure 3:**
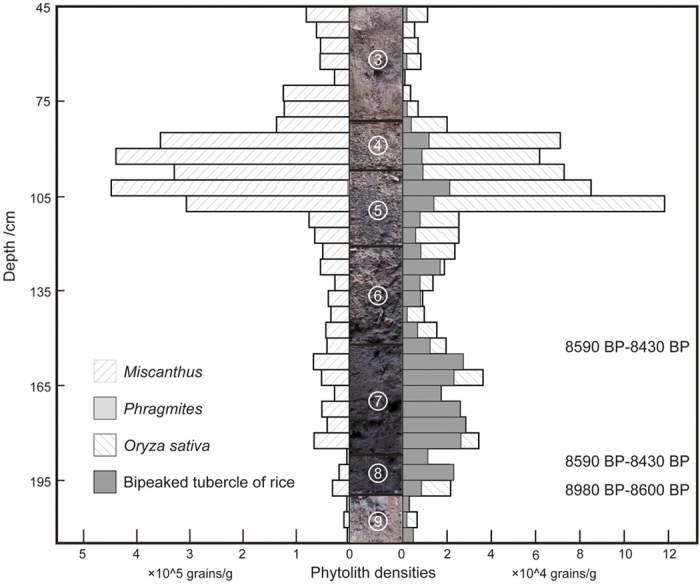
Phytoliths and their densities in the section of the south wall of ST3 trench at Huxi. Correspondence of the archaeological levels with sampled levels is indicated by the photo in the center column.

**Figure 4:**
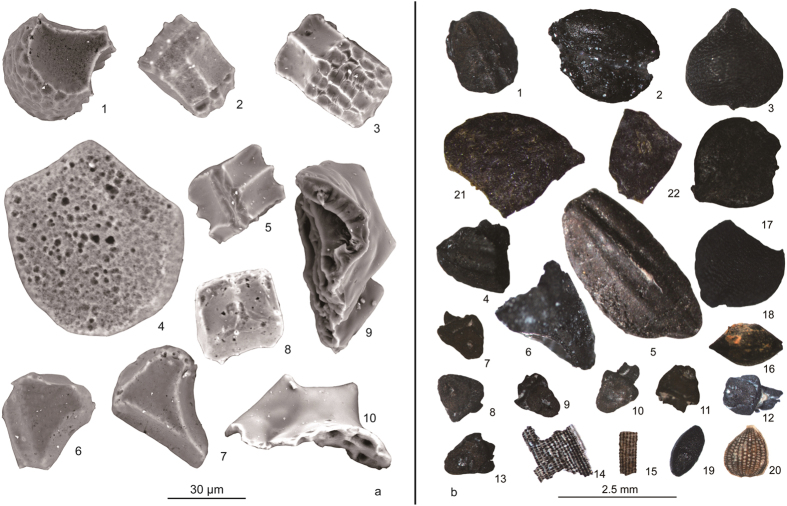
(**a**) Phytoliths from Poaceae bulliform cells (from leaf epidermis) and bi-peaked tubercles from rice husks recovered from Huxi. 1, 2, 3: rice (*Oryza* sp.); 4: reed (*Phragmites* sp.); 5: bamboo (Bambusoideae); 6, 7, 8: silvergrass (*Miscanthus* sp.); 9, 10: bi-peaked tubercles of rice. (**b**) Charred plant remains from Huxi. 1: self-heal (*Prunella* sp.); 2: cupgrass (*Eriochloa* sp.); 3, 17: sedge family (Cyperaceae); 4–6: carbonized rice; 7–13: rice rachillae; 14–15: rice husks fragments; 16: knotweed/smartweed (Polygonaceae); 18: bristlegrass (*Setaria* sp.); 19: crabgrass (*Digitaria* sp.), 20: tall fringe-rush (*Fimbristylis dichotoma*); 21, 22: foxnut (*Euryale ferox*).

Bulliform phytoliths are from rice, *Phragmites* sp. (common or ditch reed), *Miscanthus* sp. (Chinese silvergrass), and Bambusoideae (bamboos) (Figs 3 and 4a). The rice phytoliths in the cultural layers average 38693 grains/g (range: 6000 to 70000 grains/g), and 59536 grains/g in the pits (range: 20000 to 70000 grains/g). Bi-peaked tubercles average 13327 grains/g in the cultural layers (range: 800 to 25000 grains/g), and 43476 grains/g in the pits (range: 11000 to 81000 grains/g) (Supplementary Table S2). Silvergrass and rice leaf phytolith densities are positively correlated and are as much as five times higher in strata 4 and 5. In contrast, rice husk phytolith (bi-peaked tubercle) densities in the same strata are significantly less than in deeper strata (below stratum 5).

### Plant seeds and rice remains

Nine plant taxa (for scientific names see Table 1) including rice, bristlegrass, crabgrass, cupgrass, self-heal, foxnut, knotweed/smartweed, sedge family, tall fringe-rush have been identified among the 182 seeds and spikelet bases, 35 of which are seeds other than rice (Table 1, Fig. 4b). The density of the charred remains is about 4 grains/l, similar to that of the densest samples at the Early Holocene Houli culture Yuezhuang site to the north^20^. The archaeological remains of rice include 126 short rachillae (spikelet bases), some husk fragments, and a few carbonized grain fragments.

### Spikelet base morphology

Three types of spikelet bases are among the short rachillae from Huxi site (Supplementary Fig. S2): 1) a wild type with a smooth base and medullary cavity exhibiting no evidence of tearing, 2) an intermediate type with a relatively smooth base and a vascular bundle with evidence of tearing or some pedicel still present, and 3) a non-shattering type with part of the pedicel present because the spikelet was removed from the rice plant by breaking the pedicel rather than separating at the spikelet base. There are 77, 38, and 11, accounting for 61.2%, 30.1%, and 8.7% of the total, respectively.

**Figure 5:**
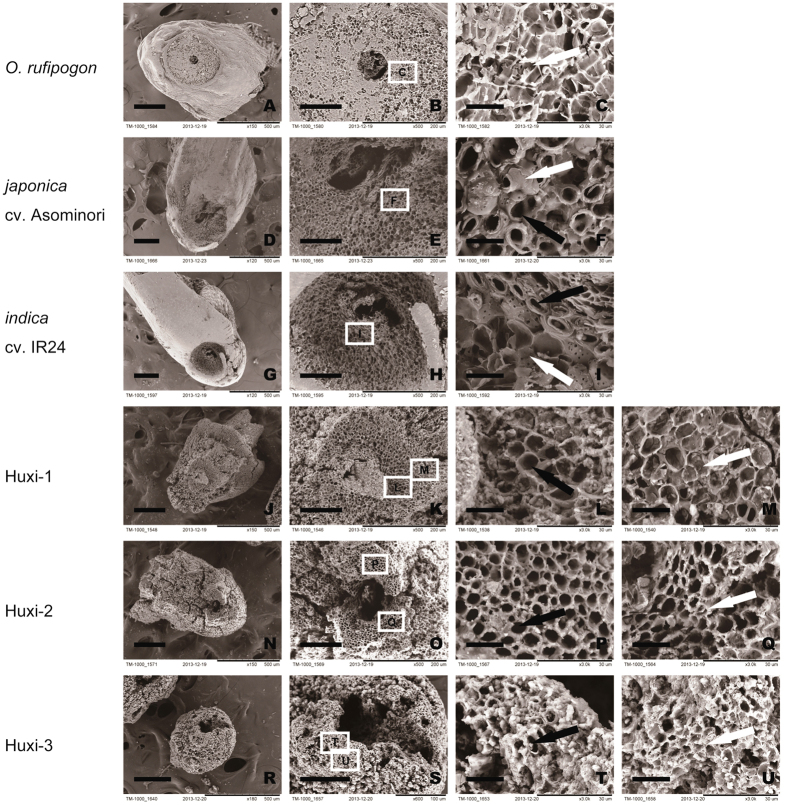
Morphological and histological comparisons of spikelet bases of modern rice and archaeological rice from Huxi site. (**A–C**) wild rice: the box in (**B**) shows the enlarged area (**C**) that is comprised entirely of abscission cells; (**D–F**) japonica rice: the box in (**E**) shows the enlarged area (**F**) with a discontinuous abscission layer; (**G–I**) indica rice: the box in (**H**) shows the enlarged area (**I**) in which the abscission layer did not develop in the region immediately surrounding the medullary cavity. The lower three rows (**J–U**) document short rachillae from the Huxi site; the boxes in (**K,O,S**) show the enlarged areas (**L,P,T**), respectively, where the abscission layer did not develop but vascular bundles formed; the boxes in (**K,O,S**) show the enlarged areas (**M,Q,U**), respectively where an abscission layer developed in some areas. The black arrows indicate where vascular bundles formed while the white arrows indicate where an abscission layer developed. Scale bar: A, D, G, J, N, R, 250 μm; B, E, H, K, O, S, 100 μm; C, F, I, L, P, T, M, Q, U, 15 μm.

SEM (TM-100, Hitachi) images (Fig. 5) show that most smooth and rough spikelet bases have incomplete abscission layers, although the abscission layers are still developed to some extent. The features differ from either wild rice with complete abscission layer development or indica rice that has incomplete abscission layer development and a few vascular bundles around a medullary cavity (pith cavity). The vascular bundles are dispersed on the spikelet bases and not as developed as in japonica rice.

## Discussion

The results of the Huxi plant remains analysis have broad implications regarding both rice domestication and its human ecological context in the Early Holocene Yangtze valley. The recovery of rice remains, especially the short rachillae of rice spikelets are particularly informative. The charred grain fragments suggest that rice was a food resource at Huxi but offer little insight into the development of domestication syndrome traits because grain size measurements are unreliable diagnostic traits^1^. Furthermore, seed size likely changes only after the domestication process is well under way. Domesticated rice has at least 20 domestication syndrome traits, most of which are quantitative and few of which can be observed in the archaeological record^1^. As a result, domestication of rice (and other grasses) is best indicated by archaeological evidence for reduced shattering that was essential for humans to efficiently harvest the crop and that rendered the plant dependent on humans^21^,^22^. Complete loss of shattering is not conducive to simple threshing methods, so relatively complete loss of shattering probably developed when better, more mechanized methods were available^23^,^24^. The considerable non-shattering variation is evidence that it is a polygenic trait, confirmed by the identification of at least 5 QTLs that are involved, two of which are considered to be the major contributors to shattering variation (qSH1 and qSH4)^18^,^25^,^26^.

The base of the spikelet in wild grasses is attached at the pedicel and is separated from the pedicel by the formation of an abscission layer comprised of parenchyma cells. Wild rice forms a complete abscission layer, whereas domesticated rice forms a variable, discontinuous layer of abscission cells between the vascular bundle and epidermis. Some varieties of japonica rice are almost completely non-shattering while some are not. Indica rice is generally more shattering than japonica because the abscission layer in indica rice does not develop in the region immediately surrounding the medullary cavity. Japonica rice on the other hand has a discontinuous abscission layer that retreats further from the vascular bundle, so it tends to be less brittle than indica^23^,^25^. The parts of the base without abscission cells have a collection of vascular bundles with pores^27^. The variation in abscission layer growth patterns among mature wild, indica, and japonica rice is, therefore, associated with spikelet base morphological differences. Wild rice has a smooth base with an intact medullary cavity, whereas, despite a smooth base, threshed indica is frequently with a torn medullary cavity. Threshed japonica rice has a rough base with some pedicel still attached, so few vascular bundles are visible. Archaeologically recovered spikelet bases thus permit distinguishing wild from domesticated rice^3^,^4^,^5^ and have the potential to diagnose whether the rice is *japonica*, *indica*, or neither.

The intermediate and non-shattering types among the Huxi spikelet bases are evidence of selection that shifted the population to reduced shattering by 9000–8400 BP. Furthermore, the Huxi spikelet bases with dispersed vascular bundles and discontinuous abscission cells contrasts with both wild and indica rice, suggesting that japonica rice was, at the very least, beginning to differentiate at the time. However, the Huxi rice with about 61 percent shattering and 30 percent intermediate had not differentiated as much as the later rice from Kuahuqiao (8000–7700 BP) where shattering (wild type) rice accounts for about 58 percent of the population there^4^. The precise ratio of shattering to non-shattering rice is disputed because some of the spikelet bases may be immature and make up a portion of the non-shattering spikelets^5^; nevertheless mature, non-shattering spikelet bases are a significant part of the Kuahuqiao assemblage in our view. Both Tianluoshan and Luojiajiao (ca.7000–6500 BP) sites, next in the cultural sequence, have 49 percent shattering rice^4^.

Differences in seed shattering between indica and japonica are controlled by five QTLs, qSH11, qSH12 in *indica*, and qSH1, qSH2 and qSH5 in japonica, all of which contribute to a decrease in seed shattering owing to the absence of abscission layer formation. The qSH1 QTL has the largest effect, explaining 68.6% of the total phenotypic variation in the population; qSH1 decreases the expression of the transcription factor only at the provisional abscission layer, resulting in reduced shattering^25^. The Huxi spikelet bases indicate that a mutation of the loss of shattering allele had already occurred. The minor alleles, qSH2 and 5 had, therefore, mutated by 8400 years ago but the major allele qSH1 had not yet.

**Figure 6:**
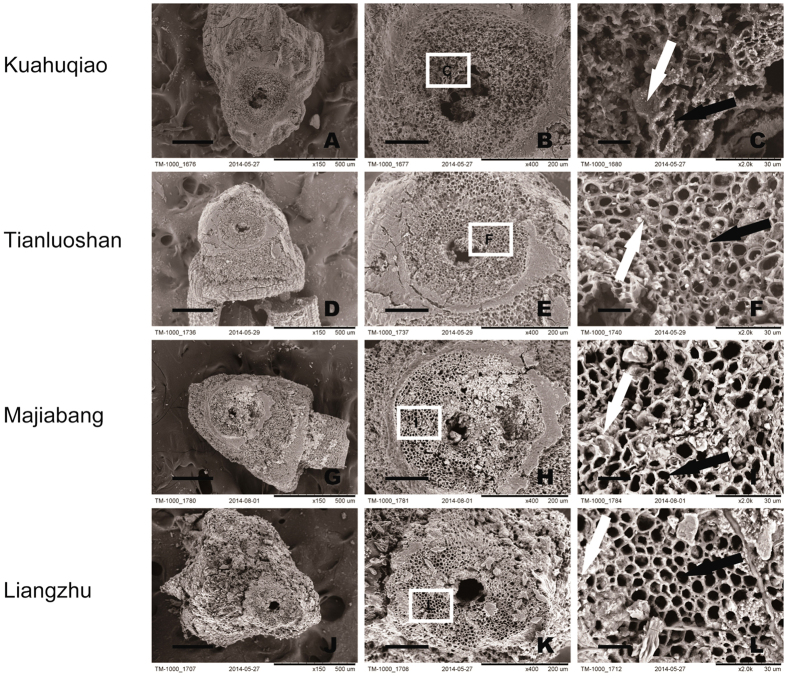
Comparison of archaeological rice spikelet bases from four sites post-dating Huxi: (**A–C)** Kuahuqiao, (**D–F**) Tianluoshan, (**G–I**) Majiabang, and (**J–L**) Liangzhu. The boxes in (**B,E,H**) and (**K**) show the enlarged areas in (**C,F,I,L**) respectively, where an abscission layer did not develop, instead vascular bundles are present. The black arrows indicate where vascular bundles formed, while the white arrows indicate where an abscission layer developed. Scale bars: A, D, G, J, 250 μm; B, E, H, K, 100 μm; C, F, I, L, 15 μm.

The spikelet bases of the Tianluoshan (7000–6500 BP) rice, and most of the specimens from Majiabang (6300–6000 BP) and Liangzhu (5300–4300 BP) sites, respectively, also have features of japonica with developed vascular bundles (Fig. 6). The vascular bundles of a few examples with smooth bases appear to have developed over time as the abscission layer became vestigial (reducing shattering). This suggests that japonica rice with the qSH1 mutation was present by at least 7000 years ago^25^ when the initial paddy system for cultivating rice was established^7^. This is supported by a DNA extracted from rice husks at Tianluoshan that links the rice to japonica^28^.

A second major QTL that affects grain shattering is shattering 4 (qSH4). This QTL is thought to be involved in cell wall degradation and/or the establishment of the abscission layer^23^,^29^. This mutation likely occurred before the differentiation of japonica and indica^30^,^31^, that is, earlier than the qSH1 mutation because it is found in both indica and japonica rice. The spikelet bases from Huxi have characteristics of japonica rice suggesting that the Huxi rice had already experienced initial selection for the non-shattering trait. Rice cultivation therefore must have begun earlier than the Huxi site occupation. This is consistent with the recent phytolith study at the Shangshan site that indicates that rice was undergoing domestication during the Shangshan period^2^ and with a molecular clock estimate based on phylogenetic sequence datasets that brackets domestication to between 13500 and 8200 BP^32^.

The domestication of cereals was a long process in general. The non-shattering phenotypes of wheat and barley, for example, gradually became fixed in cultivated populations over at least two or three millennia^23^,^33^. The spikelet bases from the Tianluoshan site, with the developed vascular bundles rather than the mostly vestigial abscission layer is evidence that non-shattering had become dominant in the cultivated populations by 7000 years ago. The evidence also suggests that the process of rice domestication is similar to that of wheat and barley. Despite higher cross-pollination rates in wild rice^34^ relative to the self-pollinating wheat and barley^35^, pollination systems may not have had an appreciable impact on the rate of domestication^5^. Instead, the presence of sympatric populations of both wild and domesticated cereals may have dampened selection for domestication^36^.

Another aspect of the record to consider is that the diffusion of an eventually important crop is an indirect indication of its economic significance and possible domestication. Some time between 9000 and 8000 BP rice grains were incorporated into the Jiahu site economy in the Huai River valley to the north of the Yangtze basin^37^ and 400 km south of Yuezhuang. The extent to which the Jiahu rice was non-shattering or outside the range of wild rice at the time is unknown. Rice grains reported from two Houli culture sites, Yuezhuang and Xihe in Shandong Province (8000–7700 BP), are evidence that rice had spread 800 km north of central Zhejiang province by the end of the Shangshan period^38^,^39^. Although evidence for rice being grown that far north (e.g. tools and ecological indicators such as weeds) is not forthcoming, rice was clearly of interest to early Neolithic people well north of the Yangtze basin suggesting that it was a plant with significant value at the time. No other evidence of a plant moving this far north from the Yangtze valley has been reported so far.

Japonica and indica rice have morphological and physiological differences, as well as significant reproductive isolation despite being grown in overlapping geographical ranges today^40^,^41^,^42^. The divergence of japonica and indica rice is not well understood, involving either separate domestications or a single domestication of *O. sativa* after which the subspecies diverged^18^,^19^,^32^. The general consensus is that rice was domesticated from a specific population with japonica affiliations and that domestication enhanced the differences^43^. In this scenario hybridization and introgression between wild and domesticated rice were responsible for the development of indica rice^19^,^43^. The characteristics of the spikelet bases of archaeological rice from Huxi are consistent with rice having been domesticated from a differentiated wild population or show the early development of domesticated japonica rice. Recent research indicates that Indochina is the source of the indica gene pool^44^. Indica resulted from the hybridization of japonica and ‘proto-*indica*’ rice after japonica was introduced to India; indica subsequently spread to Thailand during the historic period^45^.

The phytolith and seed remains complement the spikelet base data. Silvergrass and rice are the most common plant taxa among the phytoliths while common/ditch reed is quite rare (Fig. 3). The phytoliths most likely represent plants growing in, or close to, the excavated unit or washed in from nearby. Common reed requires seasonal flooding and water no more than half a meter deep when flooded so its presence suggests intermittent low-level flooding, to be expected in this region. Silvergrass is represented by four species in the region: *M. senses*, *M. floridulus*, *M. sacchariflorus* and *M. lutarioriparius*. They prefer a variety of mesic conditions, but none are as wet as common reed requires. Silvergrass is sun tolerant and prefers well-drained, rich, compacted or disturbed soil (common in anthropogenic habitats). The plants indicate that the area was seasonally wet and moist to dry. This habitat is also conducive to annual rice; that is anthropogenic habitats kept relatively free of common reed (present but rare among the phytoliths). Silvergrass may have been growing in clumps that were difficult to eradicate. Rice and silvergrass phytolith densities are directly correlated and are in high densities in levels 4, 5 and 6. The most plausible explanation is that anthropogenesis, especially rice cultivation, increased over time (see http://www.issg.org/database/species/ecology.asp?si=1121&lang=EN). We are not suggesting that paddy fields existed at Huxi, only that conditions in and near the excavated area were anthropogenic and suitable for rice.

The seeds, although not particularly abundant, are consistent with the phytolith data also providing evidence that disturbed, well-lit and dry through wetland habitats comprised the local habitats. Bristlegrass and crabgrass are common in disturbed, well-lit areas and are associated with later agricultural sites in China. Cupgrass prefers damp, disturbed areas and tends to be invasive. Foxnut and self-heal are the most common taxa other than rice. Foxnut is an aquatic plant that was an important resource at Kuahuqiao and Hemudu culture occupations. Self-heal is a perennial and a member of the mint family often used for medicinal purposes. It, too, tends to be a weedy, invasive plant. We are not aware of other reports of self-heal from archaeological contexts in China. The remaining taxa are represented by three or fewer seeds. Among them, sedges are commonly found in wetland through damp habitats. The plant assemblage is arguably evidence for a nascent Kuahuqiao-Hemudu human ecology; that is, a mixed economy that included cultivation^46^. The data demonstrate a continuous sequence of rice spikelet base abscission layer evolution (and therefore varying degrees of non-shattering rice) and niche construction/ecological engineering that began in the Shangshan Culture and continued into the later Neolithic of the region.

## Methods

### Materials

Thirty-eight sediment samples were collected from the southern section of trench ST3 at regular five cm intervals. Samples of sediment were also collected from pits for phytolith analysis. Sediment samples (1 to 18 liters) from each stratum and from the pits were wet screened to recover macro-remains (flotation was not possible due to high clay content of the sediments).

### Phytolith Analysis

Sediment samples were dried in a convection oven at 100 °C and mechanically crushed. One gram of soil and 300,000 glass beads (about 40 μm) were transferred to a 12 ml sample bottle. 10 ml of water and 1 ml of 5% sodium silicate were added, then the sample was vibrated in an ultrasonic cleaner (38 kHz, 250 W) for about 20 min to separate the particles. The sample was filtered in water to remove particles less than 20 μm in diameter, then dried again. Using the EUKITT^®^ mounting medium, the filtered sample was distributed uniformly on a microscope slide to facilitate the investigation of phytoliths. After being magnified 200 times with a Nikon E600 microscope, the phytoliths and glass beads were counted in the same field of vision (300 glass beads at least), and then the weight densities of phytoliths (no/soil wt) were calculated, according to the ratios of the phytoliths to the glasses beads.

### Wet screening Sample Analysis

Soil was placed in a 1000 ml beaker, and 500 ml of 5% NaHCO_3_was added as a dispersant. The sample was placed in a 70–80 °C water bath for 3 hours during which time the sample was stirred to separate sediment particles. The sample was then decanted through a sieve (Φ340 μm) until the water was clear. The resulting material was examined for identifiable plant remains with a stereomicroscope (Nikon SMZ1000).

### Observing rice spikelet bases

The rice rachillae were classified based on spikelet base features. The spikelet bases were sputter-coated (Hitachi E-1010) and examined using a Hitachi TM-1000 SEM. In addition, The spikelet bases from Kuahuqiao, Tianluoshan, Majiabang, and Liangzhu sites, were examined to provide comparative samples through time.

## Additional Information

**How to cite this article**: Zheng, Y. *et al*. Rice Domestication Revealed by Reduced Shattering of Archaeological rice from the Lower Yangtze valley. *Sci. Rep.* **6**, 28136; doi: 10.1038/srep28136 (2016).

## Supplementary Material

Supplementary Information

## Figures and Tables

**Figure 1:**
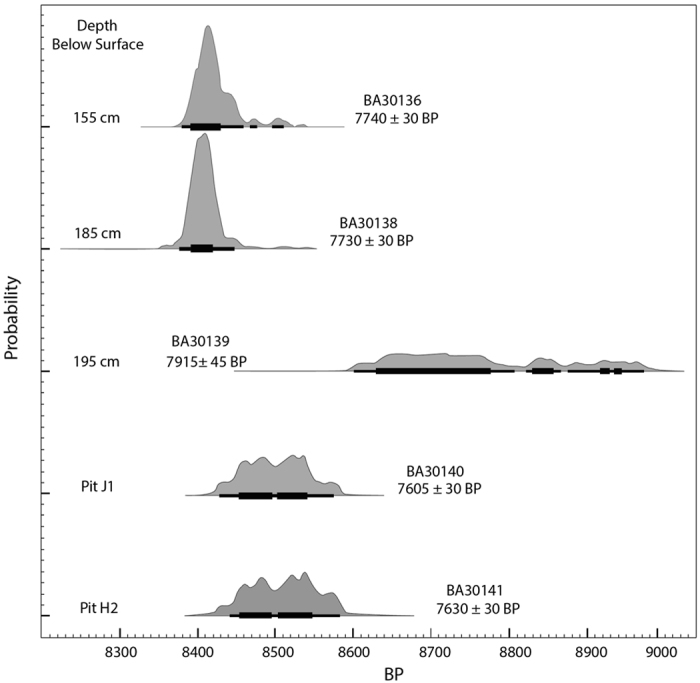
AMS Radiocarbon dates on wood charcoal from the Huxi site (Peking University AMS Laboratory, calibration by Oxcal 3.10 and INTCAL 104).

**Figure 2:**
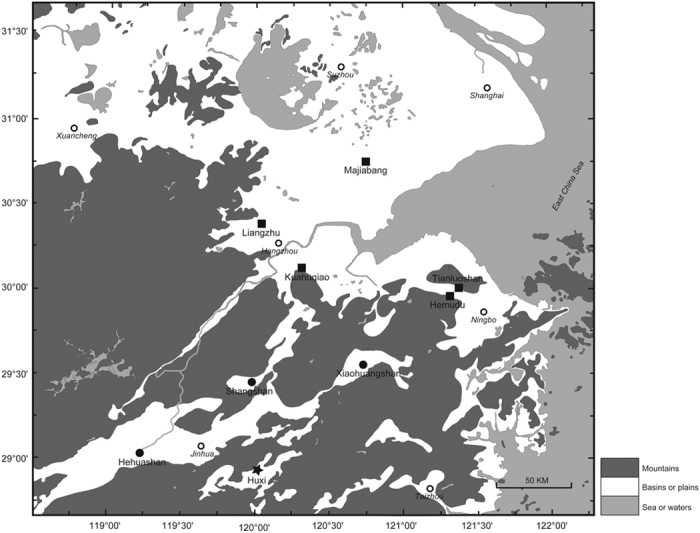
Site locations. ●, Archaeological sites older than 8000 BP; these are located in the uplands between 40 and 100 m above sea level. ■, Archaeological sites younger than 8000 BP; these are between 2 and 5 m above sea level. The map modified from Zheng *et al*.^9^, which is licensed under the Creative Commons Attribution License ( http://creativecommons.org/licenses/by/4.0/)”.

**Table 1:** Plant remains from the Huxi site.

Stratigraphic layers and pits	4	5	6	7	8	J1	H2	H7	H8	Total
Sediment volume (L)	1.5	2	2	2	1	9	18	6	6	47.5
Rice grain fragments (*Oryza* sp.)	0	0	0	0	0	1	6	0	0	7
Rice rachillae	0	1	0	0	0	24	78	0	23	126
Rice husks fragments	1	0	0	0	0	0	6	0	5	12
Bristlegrass (*Setaria* sp.)	0	0	0	0	0	1	3	0	0	4
Crabgrass (*Digitaria* sp.)	0	0	0	0	0	0	1	0	2	3
Cupgrass(*Eriochloa* sp. cf. *E. villosa*)	0	0	0	0	0	0	1	0	0	1
Self-heal (*Prunella*sp. cf. *P. vulgaris*)	0	0	0	0	0	0	1	0	0	1
Sedge family (Cyperaceae)	0	0	0	0	0	0	6	0	2	8
Tall fringe-rush (*Fimbristylis dichotoma*)	0	0	0	0	0	0	1	0	0	1
Foxnut (*Euryale ferox*)	0	0	0	2	0	2	5	0	3	12
Knotweed/smartweed (Polygonaceae)	0	0	0	0	0	1	1	0	0	2
Other	0	0	0	1	0	1	3	0	0	5
Total	1	1	0	3	0	30	112	0	35	182
Density (number/L)	1	1	0	2	0	3	6	0	6	4
