# Supplementary material for: Rice Domestication Revealed by Reduced Shattering of Archaeological rice from the Lower Yangtze valley
Source: Sci Rep. 2016 Jun 21;6:28136. doi: 10.1038/srep28136 (PMC4914946; doi:10.1038/srep28136)
Supplement: Supplementary Information [file srep28136-s1.pdf]

Rice Domestication Revealed by Reduced Shattering of Archaeological rice from the Lower Yangtze valley

Yunfei Zheng, Gary W. Crawford, Leping Jiang, Xugao Chen

SUPPLEMENTARY INFORMATION

SUPPLEMENTARY FIGURES

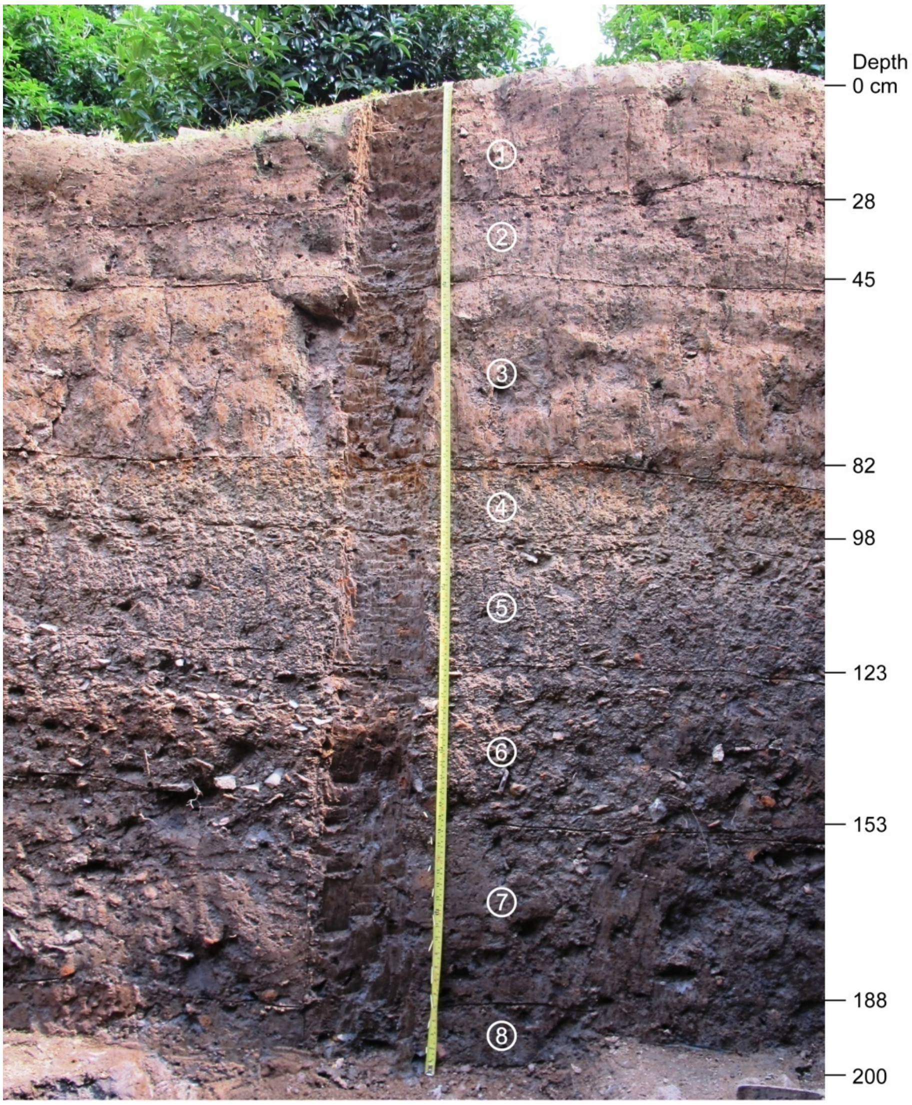

Supplementary Figure S1: Stratigraphic layers at Huxi. The southern section below the surface was divided into eight strata based on soil texture, color, and cultural inclusions: stratum 1: yellow, fine sand; stratum 2: yellow, fine sand; stratum 3: yellow, coarse silt, few earthenware fragments; stratum 4: yellow-gray; fine silt, earthenware fragments; stratum 5: yellow-gray; fine silt, numerous earthenware fragments; stratum 6: gray brown clay, a large quantity of earthenware fragments; stratum 7: gray brown clay, a small amount of earthenware fragments; stratum 8, brown clay, a small amount of earthenware fragments. stratum 3 and the following deeper are the Shangshan Culture strata.

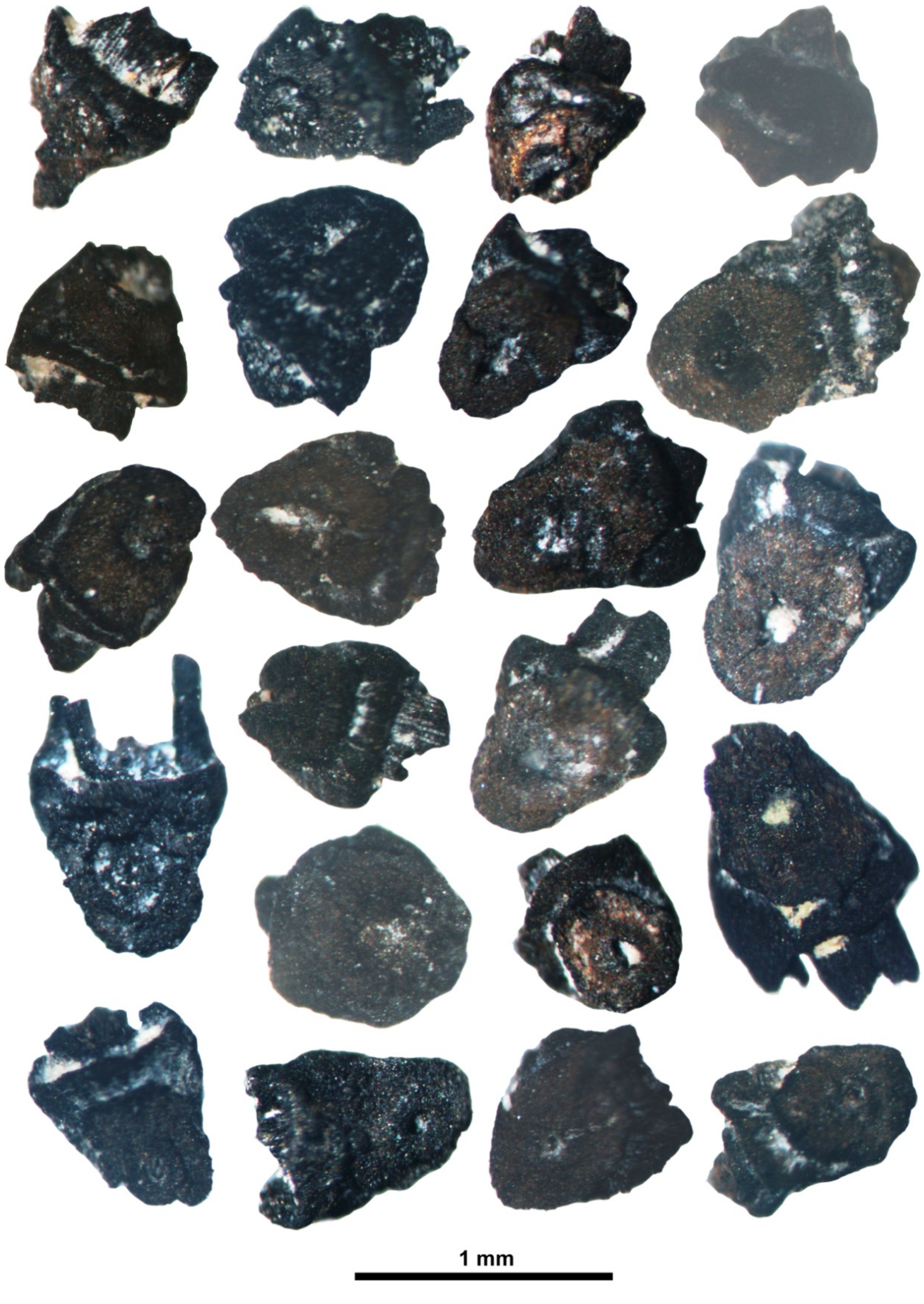

Supplementary Figure S2: Rice rachillae found in Huxi site. The spikelet bases from Huxi comprise three types: 1) a wild type with smooth base and intact medullary cavity, 2) an intermediate type with a relatively smooth base and a torn medullary cavity or with some pedicel still present, and 3) a non-shattering type with part of the pedicel retained.

SUPPLEMENTARY TABLES

Supplementary Table S1: Chronology and AMS radiocarbon dates for the Huxi site.

Strata and pits (Depth, cm, below surface)		Lab Number	Material	^{14}C age (BP, 1σ)	Calibrated age (BP, 2σ)
Stratigraphic layers	155	BA30136	Charcoal	7740±30	8510±80
	185	BA30138	Charcoal	7730±30	8510±80
	195	BA30139	Charcoal	7915±45	8790±190
Pits	J1	BA30140	Charcoal	7605±30	8410±40
	H2	BA30141	Charcoal	7630±30	8445±75

AMS, accelerator mass spectrometry, Peking University AMS Laboratory, calibrated by Oxcal 3.10 and INTCAL 104

Supplementary Table S2: Phytoliths and their densities in archaeological soil samples by archaeological strata and pits at Huxi site

Stratigraphic layers and pits	Phytolith densities grains/g				
	Oryza	*Phragmites*	*Miscanthus*	*Bambusoideae*	Bi-peaked tubercles
3	6125	260	75344	0	807
4	56441	241	315427	0	8365
5	55469	531	189226	0	11425
6	69789	697	229654	0	20361
7	26949	331	51288	310	24728
8	17384	314	18613	0	14273
Means	38693	396	146592	52	13327
J1	20905	0	29615	0	11033
H2	85981	0	71651	0	80249
H7	58444	0	50651	0	30196
H8	72816	0	43689	0	52427
Means	59536	0	48902	0	43476
